# High-Resolution Detection of Rock-Forming Minerals by Permittivity Measurements with a Near-Field Scanning Microwave Microscope

**DOI:** 10.3390/s22031138

**Published:** 2022-02-02

**Authors:** José D. Gutiérrez-Cano, José M. Catalá-Civera, Angel M. López-Buendía, Pedro J. Plaza-González, Felipe L. Penaranda-Foix

**Affiliations:** 1 Institute of Information and Communication Technologies (ITACA), Universitat Politècnica de València, Camino de Vera s/n, 46022 Valencia, Spain; jdgutierrez@itaca.upv.es (J.D.G.-C.); pedplago@itaca.upv.es (P.J.P.-G.); fpenaran@dcom.upv.es (F.L.P.-F.); 2 CEINNMAT Innovaciones, INNCEINNMAT, SL, C/Catedrático Agustín Escardino, 9, 46980 Valencia, Spain; angel.lopez@ceinnmat.com

**Keywords:** rock-forming minerals, microwave imaging, near-field scanning microwave microscope, permittivity

## Abstract

The identification of the minerals composing rocks and their dielectric characterization is essential for the utilization of microwave energy in the rock industry. This paper describes the use of a near-field scanning microwave microscope with enhanced sensitivity for non-invasive measurements of permittivity maps of rock specimens at the micrometer scale in non-contact mode. The microwave system comprises a near-field probe, an in-house single-port vectorial reflectometer, and all circuitry and software needed to make a stand-alone, portable instrument. The relationship between the resonance parameters of the near-field probe and the dielectric properties of materials was determined by a combination of classical cavity perturbation theory and an image charge model. The accuracy of this approach was validated by a comparison study with reference materials. The device was employed to determine the permittivity maps of a couple of igneous rock specimens with low-loss and high-loss minerals. The dielectric results were correlated with the minerals comprising the samples and compared with the dielectric results reported in the literature, with excellent agreements.

## 1. Introduction

Identifying the minerals that constitute rocks is a fundamental task in earth sciences, as well as in other engineering and industrial applications. Some techniques can only identify the type and amount of the minerals comprising the rock. However, many techniques can define the position of the heterogeneously distributed minerals within the structure. Particularly noteworthy are polarized light microscopy, X-ray diffraction (XRD), or X-ray fluorescence (XRF) [1,2,3]. The scientific literature reveals a large number of alternative techniques for this identification process, such as laser-induced breakdown spectroscopy (LIBS), scanning electron microscope supported with energy dispersed spectroscopy (SEM/EDS), or an electron probe microanalyzer (EPMA) [4,5,6,7]. In addition, other studies report techniques or algorithms, such as image treatment or neural networks, for the automatic identification of minerals, based on the information retrieved using some of the techniques mentioned above [8,9,10,11,12,13].

In recent years, there has been a growing interest regarding the utilization of microwave energy as a clean, green, and sustainable methodology for treating materials [14,15,16]. In the rock industry, microwave energy can contribute to developing more efficient, fast, and eco-friendly processes in various applications, such as fracture, cutting, comminution, leaching, or natural stone processing [17,18,19,20,21,22].

Complex permittivity describes the materials’ behavior in the presence of an electromagnetic field; therefore, it is one of the most relevant parameters to study in a high-power microwave application process [23].

Dielectric characterization provides the absorption capabilities of the rocks and, hence, is directly related to the heating patterns. Likewise, permittivity data provides the needed information for the design of microwave applicators for a specific high-power application.

Permittivity measurements can be classified in a large number of techniques [24,25,26,27,28,29], which can be categorized according to the measurable frequency range and the attributes of the materials to be characterized, such as dielectric losses, shape, or homogeneity [30]. Since rocks are heterogeneous materials, the approaches followed to measure their dielectric properties are very varied. 

Some authors have measured the bulk dielectric properties of a certain unidentified volume of the complete rock specimen; hence, the permittivity obtained is a mixture of the permittivity of the different minerals comprising the rock. For instance, Lu et al. [31] measured the permittivity of basalt, gabbro, and granite rocks employing the coaxial transmission line technique, shaping the rocks in the shape of a tube (7 mm outer diameter, 3 mm inner diameter, and 10 mm length) to fill the coaxial airline. Deyab et al. [32] reported the dielectric properties of kimberlite and granite samples through the coaxial probe technique, which retrieves the permittivity of a considerable region surrounding the measurement tip. Similarly, Lovás et al. [33] determined the permittivity of several minerals, such as andesite or magnesite, by a resonance cavity method using rod samples (3 mm diameter and 12 mm length). In addition, some authors have developed methods to employ the bulk changes in dielectric properties (and thus in the resonant parameters) to identify physical changes in building materials [34,35].

On the other hand, some authors measured the permittivity of the homogeneous rock-forming and accessory minerals, in pure form, isolated from the rocks. For instance, Zheng et al. [36] employed a customized rectangular resonant cavity to determine the dielectric properties of pulverized high-grade minerals, removing the air influence by means of the complex refractive index (CRI) equation. However, with this approach, as rocks are heterogeneous materials, rock-forming materials should be identified, located, isolated, and measured.

Other authors have studied the microwave-absorbing capabilities of rocks and minerals, running heating tests with domestic or industrial microwave systems and analyzing the material behavior over time [19,37]. Accordingly, there is a lack of permittivity measurement techniques that fit the requirements of heterogeneous rock specimens in the scientific literature. Near-field scanning microwave microscopes (NSMM) can fill this gap, as they can achieve permittivity maps of heterogeneous planar materials.

Near-field scanning microwave microscopes (NSMM) are measurement devices able to determine the electromagnetic response of materials on length scales far shorter than the wavelength of the emitted signal [38]. To accomplish near-field radiation, the size of the microwave probe (D) must be smaller than the probe-to-sample distance (r), and both parameters must be far shorter than the wavelength (λ) of the transmitted signal (D ≤ r << λ), achieving spatial resolutions on the range of D. The fundamental element of an NSMM is the near-field microwave probe, which is frequently implemented by a sharp coaxial aperture [39,40]. Additionally, many scientific works explore alternative geometries for that purpose, such as spiral resonators or open-ended circular waveguides [41,42]. An NSMM also comprises a microwave emitter and receptor, usually a vector network analyzer (VNA), and a positioning system [43].

Microwave probes can be configured as resonant or non-resonant structures [38]. A resonant configuration increases sensitivity but decreases measurement bandwidth [44]. Non-resonant configurations relate the changes in the magnitude and phase of the reflection or transmission coefficients to the physical changes of materials. On the other hand, resonant setups employ the shifts in the resonant frequency and quality factor parameters to determine the physical properties of the materials. The relationship between the measurable quantities and the physical parameters can be addressed through several techniques, such as full-wave analysis or lumped-element circuit models [45,46]. The cavity perturbation technique has also been widely used, since the electromagnetic fields inside the sample are much smaller than the fields in the resonator. The theory developed by Gao and Xiang [47] is one of the most used approaches for quantitative microscopy of complex permittivity [40,48,49,50].

Nowadays, the trend of NSMM devices is moving towards achieving nanometric resolutions, which requires a precise distance-following technique usually performed by means of an additional nanoscale microscope technology, such as scanning tunneling microscopes (STM) or atomic force microscopes (AFM) [51,52,53]. NSMMs have been used in a broad range of applications, such as dielectric microscopy of substrates, defects identification, or even biological cells imaging [54,55,56]. Monti et al. [57] already employed a contact-mode NSMM device with sub-micrometric resolution to elucidate the underlying physical processes that control the microwave heating of rocks at a fundamental level of some micrometric hematite inclusions in a gangue matrix (around 10 µm x 10 µm size).

In a previous work [58], we demonstrate the benefits of the NSMM technology at the micrometer scale to identify markers on anti-counterfeiting applications. Similarly, the development of an NSMM device able to determine permittivity maps at the sub-micrometric scale without contact between the sample and the probe would improve the applicability of this technology in the rock industry for predicting the heating patterns of samples to be processed under microwave irradiation. Additionally, this device would be interesting to use as an automated system to reduce times in other industrial applications, such as drill core logging, widely used for geological exploration in mining or geotechnics, for lithology, mineral identification, or structures identification [59,60,61].

In this work, we describe the use of near-field microwave microscopy in obtaining contactless complex permittivity scans of rocks at a sub-millimeter scale. For that purpose, the microscope probe reported in [39] was replaced with a larger probe with a redesigned tip, increasing the sensitivity of the near-field probe for loss factor measurements to fit the specific requirements of this application. The methodology to calculate permittivity values from resonant measurements reported in our previous work was also improved by a combination of classical cavity perturbation theory and an image charge model. The NSMM sensor was employed to determine the permittivity maps of some representative specimens of ornamental rocks and compare the permittivity results with those reported in previous works. 

## 2. Materials and Methods

### 2.1. The Near-Field Microwave Microscope

The sensing experiments were carried out in a near-field microwave microscope similar to the system reported in [58] with specific modifications to measure rocks.

**Figure 1:**
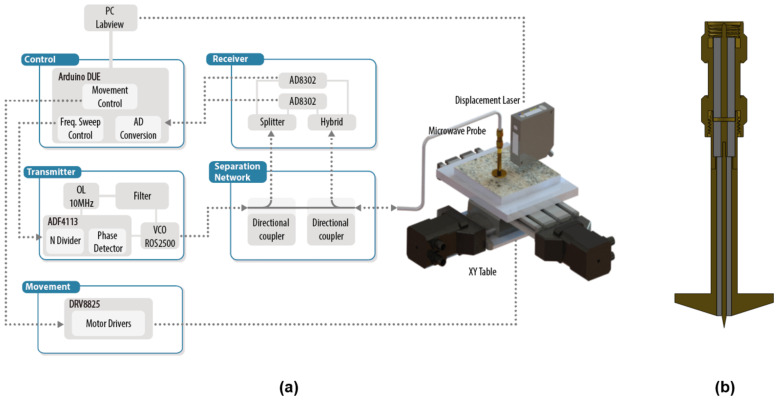
Schematic diagram of the near-field microwave microscope: (**a**) schematic diagram of the in-house vector network analyzer for measuring the reflection (S_11_) of the near-field microwave microscope; (**b**) cross-section of the near-field coaxial resonator.

Figure 1 illustrates a schematic diagram of the different parts that constitute the instrument (near-field microwave probe, microwave source and detector, and positioning subsystem) with a zoomed image of the upgraded sensor probe.

The measurement probe consisted of a capacitively fed resonant coaxial cavity with an inner conductor, enlarged and tapered to meet the requirements for near-field radiation (D ≤ r << λ). The response of the probe around the resonance peak S_11_ contains all the information required to determine the permittivity of the material. 

In this work, the dimensions of the measurement probe were larger than those in our previous design to increase the sensitivity of the quality factor response and thus the resolution in the loss factor measurements. The size of the resonant coaxial line corresponds to the size of a commercial RG402 line, with a sharp tip of ~50 µm radius. The length of the coaxial line was 53.5 mm to achieve an air resonance frequency close to 2 GHz. Since the study’s main objective is to predict the heating patterns of samples to be processed with high-power microwave energy, we employed a resonant frequency value between 915 MHz and 2.45 GHz, the most common frequencies to process materials under high-power microwave irradiation [62].

**Figure 2:**
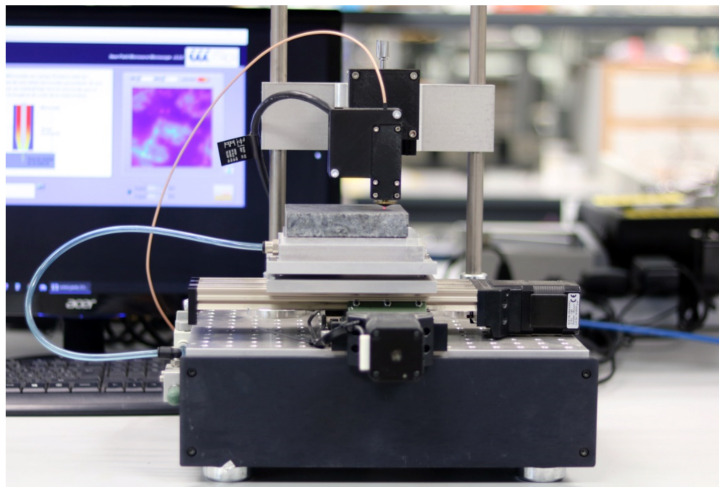
The near-field microwave microscope system.

The microwave source and detector were based on an in-house, single-port vectorial reflectometer design, described in [30] (see Figure 2). The microwave source is a frequency synthesizer based on a phase-locked loop (PLL), comprising an external filter, a local oscillator (OL), a voltage-controlled oscillator (ROS-2500, Mini-Circuits, Brooklyn, NY, USA), and an ADF4113 (Analog Devices, Norwood, MA, USA), which includes the divider and the phase detector. The reflection parameter or S_11_ is calculated at the receiver by comparing the PLL’s incident signal with the signal reflected at the probe. In this regard, a separation network, comprising two bi-directional couplers (BDCA 1-7-33 +, Mini-Circuits), was employed previously to collect a small amount of those incidents and reflected signals. The receiver was based on the AD8302 RF/IF Gain and Phase Detector (Analog Devices). Since the phase response of this integrated circuit is similar for positive or negative phase differences, two AD8302 units were needed to solve the phase ambiguity (see [33] for further information).

The positioning subsystem comprised an XY-stage (KT-70, proxxon) with a vacuum table attached, driven by two stepper motors and drivers (DRV8825, Texas Instruments, Dallas, TX, USA). The probe was attached in a fixed position over the XY-stage, manually positioned by a micrometric screw. In addition, a displacement laser (HL-G103-SJ, Panasonic, Osaka, Japan) was utilized to precisely determine the tip-to-sample distance. An Arduino board, together with a Labview piece of software running in a computer, control all the elements of the microscope: generating the incident signal, receiving the reflected signal, calculating the reflection coefficient and the resonant parameters, calculating the dielectric properties, plotting the results, moving the XY-stage, and synchronizing the movements with the measurements. Figure 2 depicts the stand-alone NSMM system. 

The single-port vectorial reflectometer was allowed to stabilize for one hour before measurements. Subsequently, a standard OSM calibration procedure was carried out from 1930 to 1970 MHz employing the 85052 B Standard Mechanical Calibration Kit (3.5 mm, Keysight Technologies, Santa Rosa, CA, USA).

### 2.2. Dielectric Characterization

As described in Section 2.1, the near-field sensor was configured as a resonator; thus, the permittivity is related to the resonance frequency (fr) and the quality factor (Q) of the microwave structure. The fr and unloaded Q values were calculated from the reflection coefficient measurements through the linear, fractional curve-fitting procedure reported by Kajfez [63,64]. 

**Figure 3:**
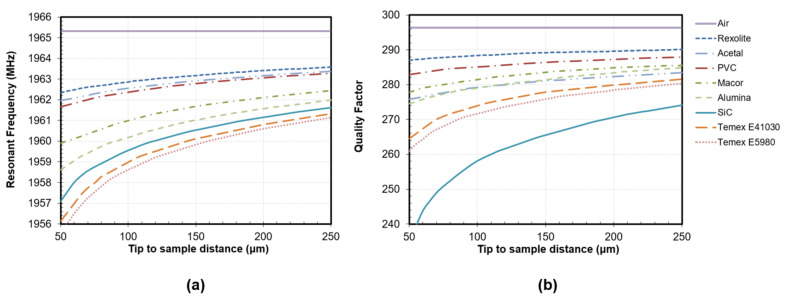
Response of the sensor as a function of the tip-to-sample distance for different reference materials: (**a**) resonant frequency; (**b**) unloaded quality factor.

Figure 3 shows the response of the near-field resonant probe (fr and Q) for a set of rod-shaped materials as a function of the tip-to-sample distance (g). These materials were selected to cover a wide range of both dielectric constant (ε′) and loss factor (ε″) values, with a sample size sufficient to be considered as infinite dielectric materials (15 mm height, 9.8 mm diameter) [40,65]. The fr curves exhibited similar trends to those shown by the smaller probe reported in our previous work [58]. Nevertheless, the Q sensitivity increased substantially due to the improvements.

The operating range of the probe was centered between 50 µm and 250 µm, seeking a balance between the probe’s sensitivity and the robustness against uncertainties in the determination of g. Below 50 µm, the sensitivity of the probe would increase remarkably, but the uncertainties in the determination of g would lead to significant uncertainties in the retrieved permittivity results. In addition, the minor imperfections in the surface of the materials and the non-horizontality of the base could cause the probe tip to touch the material and be damaged. Above 250 µm, the probe’s response would be more robust against errors in the determination of g, but the sensitivity of both fr and Q parameters would decrease.

All microwave scans were performed at a central g of 150 µm. Nonetheless, the permittivity model should include the influence of the tip-to-sample distance to account for possible variations of this parameter during the scan because of irregularities in the height of the material and uneven horizontality of the material surface when placed over the microscope’s base. At 150 µm, the maximum fr shift, presented between air (ε′= 1) and Temex E5980 (ε′ = 67.39), was around 6 MHz. The response of the probe compresses as the dielectric constant of the MUT increases; thus, the fr shift between air (ε′= 1) and Rexolite (ε′= 2.57) is similar to the shift observed between Rexolite and Macor (ε′= 5.68), and higher than the deviation exhibited between alumina (ε′= 9) and Temex E5980 (ε′ = 67.39). Regarding the behavior of the quality factor, the maximum deviation, observed between air (ε″ = 0) and SiC (ε″ = 2.07), was around 30. However, it is noteworthy that, for a given g, the Q of materials with similar loss levels decreases as ε′ increases (fr decreases). Thus, Rexolite (ε″ = 0.0008, tan δ = 3 × 10^−4^) and Temex 41030 (ε″ = 0.0007, tan δ = 2∙10^−5^), materials with similar dielectric losses, have considerably divergent Qs, 289.17 and 277.85, respectively: for a given loss factor, the resonance widens with increasing dielectric constant.

In our previous work [58], the determination of the dielectric properties was addressed using the microwave cavity perturbation technique (MCPT). In the near-field zone, with evanescent waves, the field distribution can be considered as a static problem where phase and retardation need not be considered [50]. Then, assuming a quasi-static electric field inside the MUT [66], we employed Khanna et al. [67] to relate the shifts in fr and Q with permittivity. For convenience, we reproduce the below formulas:

(1) $$\varepsilon^{\prime }=1+\frac{-\frac{\Delta f}{f}\left(\eta +N\frac{\Delta f}{f}\right)-N{\left[\Delta \left(\frac{1}{2Q}\right)\right]}^{2}}{{\left(\eta +N\frac{\Delta f}{f}\right)}^{2}+N^{2}{\left[\Delta \left(\frac{1}{2Q}\right)\right]}^{2}}$$

(2) $$\varepsilon^{\prime \prime }=\frac{\eta \Delta \left(\frac{1}{2Q}\right)}{{\left(\eta +N\frac{\Delta f}{f}\right)}^{2}+N^{2}{\left[\Delta \left(\frac{1}{2Q}\right)\right]}^{2}}$$

(3) $$\frac{\Delta f}{f}=\frac{f_{s}-f_{0}}{f_{s}}$$

(4) $$\Delta \left(\frac{1}{2Q}\right)=\frac{f_{0}}{f_{s}}\cdot \frac{1}{2}\cdot \left(\frac{1}{Q_{s}}-\frac{1}{Q_{0}}\cdot \frac{f_{s}^{2}}{f_{0}^{2}}\right)$$

where *ε*′ = dielectric constant (dimensionless); *ε*″ = loss factor (dimensionless); *η* = sample-filling factor (dimensionless); *N* = sample-depolarization factor (dimensionless); *f*_0 =_ resonant frequency (Hz) of the open-air cavity; *f_s_* = resonant frequency (Hz) with a dielectric material near the tip; *Q*_0_ = quality factor (dimensionless) of the open-air cavity; *Q_s_* = quality factor (dimensionless) with a dielectric material near the tip. 

**Figure 4:**
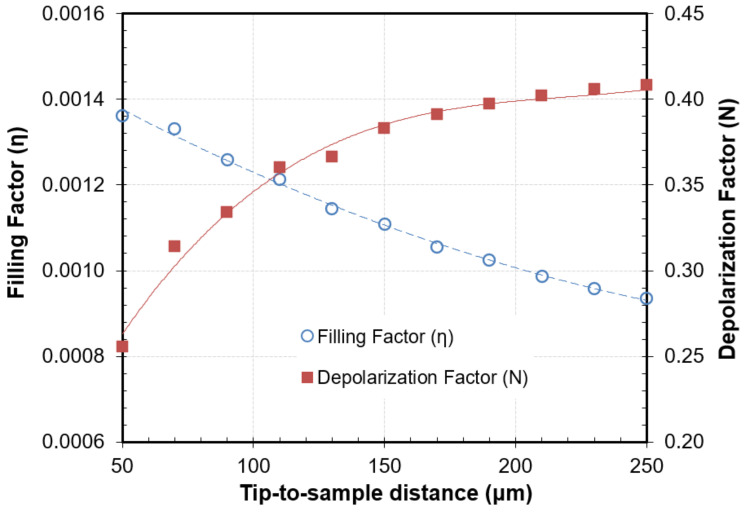
Calibration parameters, η and N, of the cavity perturbation method determined with reference samples as a function of the tip-to-sample distance.

However, this model did not conform precisely to the response measured with the higher size probe employed in this work, mainly due to the aforementioned widening of the resonance with the increase in the dielectric constant. However, it is well known from the literature that the determination of the dielectric constant in NSMM measurement systems is independent of the Q changes (see Equations (5) and (6)). Hence, the parameters η and N needed to determine the dielectric constant values from the fr measurement as a function of g can be obtained by removing Q’s influence (Q_s_ = Q_0_) on Equation (1) (see Figure 4). To obtain these curves, three materials were employed: air, Macor (ε′ = 5.68), and Temex E41030 (ε′ = 28.28). However, this model (Equation (2)) did not accurately reproduce all the dielectric losses of the analyzed materials.

Considering quasi-static fields, Gao and Xiang [47] developed a model for the case of a conducting spherical tip radiating over a dielectric material employing an image charge model and the MCPT. The relationship between the dielectric constant and the fr shift for the case of soft contact (g = 0) was determined as follows:

(5) $$\frac{\Delta f}{f_{0}}=-A\left(\frac{\ln(1-b)}{b}+1\right)$$

where *b* = (ε′ − 1)/(ε′ + 1), and *A* is a constant to be minimized, related to the geometry of the near-field probe. For our specific case, where there is an air gap between the dielectric sample and the probe’s tip (g > 0), the equations became an iterative problem, as follows:

(6) $$\frac{\Delta f_{s}}{f_{s}}=-A\sum_{n=1}^{\infty }\frac{bt_{n}}{{a^{\prime }}_{1}+{a^{\prime }}_{n}}$$

(7) $${a^{\prime }}_{n}=1+a^{\prime }+\frac{1}{1+a^{\prime }+{a^{\prime }}_{n-1}},t_{n}=\frac{b}{1+a^{\prime }+{a^{\prime }}_{n-1}}t_{n-1}$$

where Δ*f_s_* = *f_s_* − *f*_0_. The initial conditions were as follows: *a*′ = g/Ro, being g the tip-to-sample distance and R_0_ the tip radius; *a*′_1_ = 1 + *a*′; and *t*_1_ = 1. For both configurations, the dielectric losses were determined to be related to the fr and *Q* shifts, as follows:

(8) $$\Delta \left(\frac{1}{Q}\right)=\frac{1}{Q_{s}}-\frac{1}{Q_{0}}=-(B+\tan\delta )\frac{\Delta f_{s}}{f_{s}}$$

where *B* is a constant to be minimized and tan*δ* = ε″/ε′. Over the years, some scientific works have tried to improve the accuracy of this model, especially concerning dielectric losses, adding new constants or quadratic terms to Equation (8) [48,49,50]. Finally, Gregory et al. [40] reported the use of Equation (6) with the complex resonant frequency (f_s_* in Equation (9)) to avoid the use of Equation (8).

(9) $$f_{S}{}^{*}=f_{S}\left(1+\frac{j}{2Q}\right)$$

**Figure 5:**
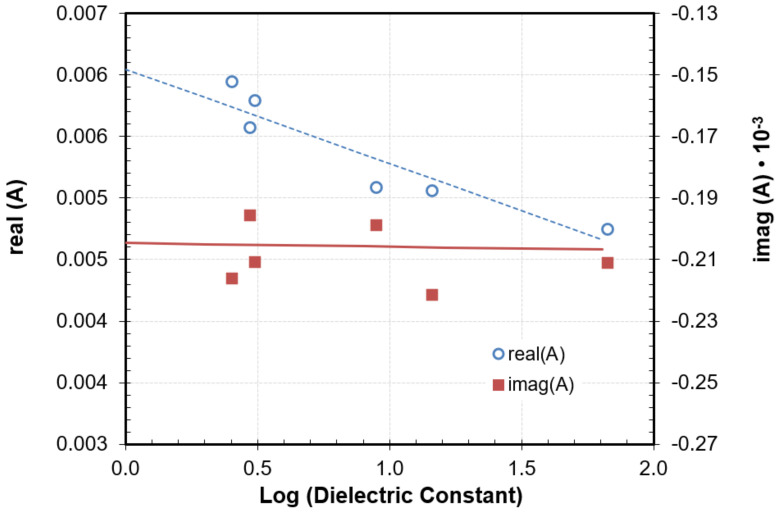
Calibration parameter A of the image charge model determined with reference samples as a function of the dielectric constant.

The increase in the size of the probe and the geometry of this tip has allowed the use of Equation (6) with the complex resonance parameter (Equation (9)) to model the response of this new probe with the improved sensitivity of Q. However, the parameters minimized showed certain dependence with frequency or dielectric constant, as reported in previous works [48,49] for the parameters determined in the dielectric loss equation. Figure 5 shows the dependence of *A* with the logarithm of the dielectric constant of some reference materials (Rexolite, Acetal, PVC, Alumina, SiC, and Temex E5980). This result would preclude the use of this method to calculate the complex permittivity from the relative shifts of fr and Q in our case, since it would require prior knowledge of the dielectric constant value of the measured material. To overcome this limitation, Equation (1) (with Q_s_ = Q_0_) was employed with the parameters interpolated in Figure 4 to determine the dielectric constant values. This value was employed to determine A from Figure 5, and the loss factor values were calculated using Equation (6) with the complex resonant frequency concept (Equation (9)).

### 2.3. Rock Samples

Two rock specimens were selected to test the proposed microscopy technique. The selected rocks, representing felsic and mafic families, are used widely in ornamental material construction as natural stones.

**Figure 6:**
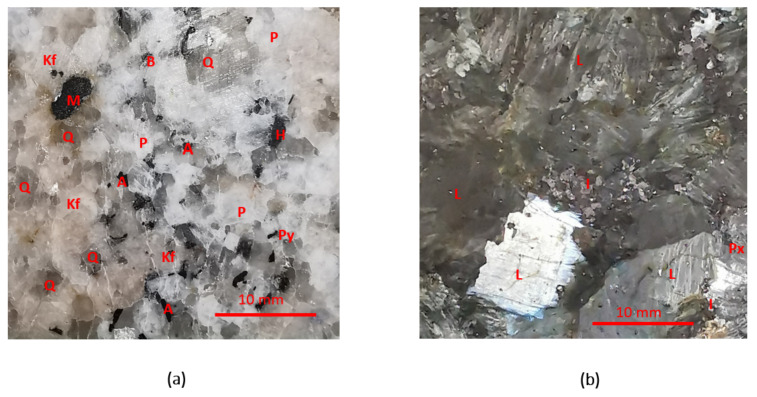
Microscope images of two types of rock: (**a**) gneiss (Q—quartz; Kf—potassium feldspar; P—plagioclase; B—biotite; A—aegirine-augite pyroxene; H—hornblende; M—magnetite; Py—pyrite); and (**b**) anorthosite (L—labradorite plagioclase; Px—pyroxene; I—ilmenite).

Rocks pieces were cut in sections of 10 × 10 × 2 cm with the surface polished to improve the readability of the system. The mineral composition of the rock specimens depicted in Figure 6 was determined by petrographic polarized light microscopy, chemical microanalysis EDX in a scanning electron microscope (SEM), and X-ray analysis of the bulk sample and the magnetic fraction. Table 1 summarizes the dielectric properties references of the rock-forming minerals found in this analysis.

The felsic rock selected was a high-grade granitic gneiss. The analysis of the rock identified quartz, potassium feldspar (microcline), and alkaline plagioclase as major components, pyroxene (aegirine-augite) and biotite as secondary, and associate minerals represented by amphibole (hornblende), tourmaline, and opaque minerals. These opaque minerals were mainly magnetite (Fe_3_O_4_) (that appeared in aggregates with apparent inter-crystalline porosity and occasionally pyrrhotite (S_x_Fe_1-x_)-associated) and dispersed pyrite (FeS_2_).

The mafic rock selected was anorthosite, a peralkaline gabbro with blueish and greenish-grey color. The rock was composed massively by macroscopic Na–Ca plagioclase of labradorite type domain and minor content of pyroxene and biotite. In addition, the rock was composed by a significant amount of oxide ores, mainly ilmenite (FeTiO_3_) with some magnetite (Fe_3_O_4_) associated.

## 3. Experimental Results and Discussion

### 3.1. Dielectric Measurements of Reference Materials

The complex permittivity of the reference samples, employed to determine the response of Figure 3, were measured to verify the accuracy of the procedure described in Section 2.2. Each dielectric sample was positioned on the microscope base centered in the axis of the near-field probe. From the point of soft contact, the Z-axis was moved upwards to three different tip-to-sample distances (100 µm, 150 μm, and 200 µm), measuring the reflection parameter employing the reflectometer and determining the resonance parameters, fr and Q. The parameters N and η corresponding to each g were determined to calculate the dielectric constant of the measurements. With the results obtained, the appropriate *A* parameter was interpolated to obtain the loss factor. All measurements were retrieved at a room temperature of 23 °C (see Table 2). 

To assess the accuracy of the results, the permittivity of the reference materials was also measured in a closed TM_010_ cylindrical cavity (98 mm diameter, 20 mm height) analyzed by the mode-matching technique described in [74]. The error (measurement bias), also reported in Table 2, provides a complete image of the device’s performance and corresponds to the absolute difference between the measured mean value and the reference value. The reported standard deviation values provide information mainly related to the uncertainties in the height positioning and the stability of the electronics.

The determination of the dielectric constant revealed an excellent accuracy, with errors constrained below 3%, in comparison with the reference values.

The standard deviation values also exhibited little variability as a function of the tip-to-sample distance. However, the error increased slightly with the dielectric constant increment due to the compression in the resonant frequency response discussed in Section 2.2.

From the discrepancies observed in the results concerning the reference values, the accuracy of the loss factor measurement was below 10% within the range from 10^−2^ to 10^1^, a noticeable result considering the large tip-to-sample distances employed in this work. Below 10^−2^, even though the percentage errors were above 10%, the system is valid to determine whether it is a low-loss or a high-loss material. The standard deviation of the loss factor values revealed low figures with g variations for most materials. However, the compression effect of Q shifts was noticeable for high dielectric constant materials. For instance, the loss factor results calculated for Temex 5980 revealed fluctuations between 10^−3^ and 10^0^. Nevertheless, it is important to highlight that the common use of the loss tangent definition (ε″/ε′) would mitigate this issue.

### 3.2. Permittivity Maps of Rock Specimens

The rock specimens were placed on the base of the microscope and fixed with the aid of the suction vacuum table. The tip-to-sample distance was set to 150 µm by means of the displacement laser and the Z-axis stage. Microwave scans explored a surface area of 33 mm × 33 mm with a spatial step of 100 μm. For each point of the surface scan, the dielectric properties were calculated from the information collected from the microwave reflectometer and the displacement laser, using the approach described in Section 2.2. All measurements were performed at a room temperature of 23 °C. 

#### 3.2.1. Gneiss

**Figure 7:**
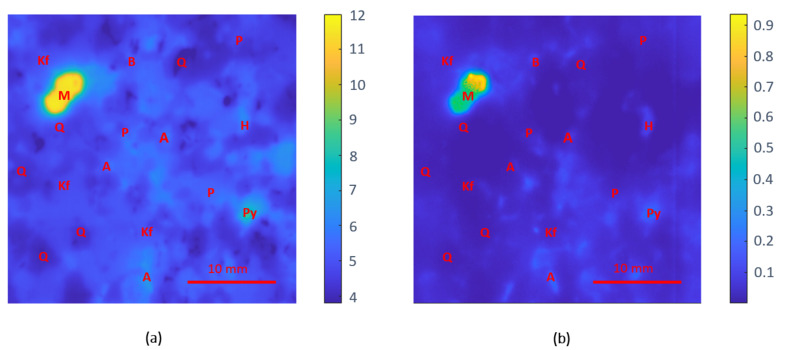
Dielectric images of the gneiss specimen: (**a**) dielectric constant response and (**b**) loss factor results (Q—quartz; Kf—potassium feldspar; P—plagioclase; B—biotite; A—aegirine-augite pyroxene; H—hornblende; M—magnetite; Py—pyrite).

Figure 7 shows the dielectric mark of the gneiss rock specimen. The dielectric pattern of both dielectric constant and loss factor results was very similar, agreeing with the mineral classification reported in [36]. Figure 7a shows a large area with moderate dielectric constant values ranging between 4 and 6 and a specific area in the left top corner, in which the values were above 10. Concerning the loss factor response, Figure 7b shows that most rock areas had low loss factor values and, therefore, had a reduced ability to absorb electromagnetic energy. However, other small regions disseminated throughout the specimen did exhibit moderate or high loss factor values.

From the petrographic analysis shown in Figure 6a, it was possible to correlate the rock’s mineral composition with the measured permittivity. First, we found a large quantity of quartz mineral, a material commonly employed as a microwave inert element for microwave processing applications [75]. In these areas, the dielectric constant was around 4 with loss factor values below 10^−2^, which indicated the low capacity of quartz to absorb microwave energy as exhibited in the heating experiments reported in [76] and in other related reports [70]. The petrographic analysis also determined a significant presence of K-feldspars (microcline). In these areas, the permittivity obtained was between 4 and 5 for the real part and in the range of 10^−2^ for the imaginary part, with the results agreeing well with those reported in [68,69]. 

Sorting the materials by their permittivity results, the area with dielectric constant values between 5 and 6 and loss factor in the range from 10^−2^ to 0.1 corresponds to the biotite and plagioclase content. These results agree closely with those obtained in [70,71] and differ from those reported in [37]. Following, we found the pyroxene aegirine-augite and hornblende content, with dielectric constant results ranging from 6 to 7 and dielectric losses between 0.1 and 0.2. These results agree well with the permittivity reported of augite pyroxene in [36]. Again, the data reported in the literature provides dissimilar results for hornblende, with dielectric constant values ranging from 7 to 14.45 and loss factor values ranging from 0.02 to 0.32 [36,37,71]. Nevertheless, the dielectric loss of pyroxene and hornblende appears to be higher than those of the surrounding materials (quartz, feldspar, biotite, or plagioclase) since the content of this mineral is easily distinguished in the loss factor map. However, regarding the dielectric constant, the calculated values were lower than those in the literature, probably due to the penetration capabilities of electromagnetic waves at microwave frequencies [58] and the small size of the crystals of these minerals in this rock specimen observed in the petrographic analysis of the sample.

Finally, we can find the oxide ores magnetite and pyrite. The complex permittivity calculated for the aggregated magnetite content, located in the left top corner, was close to 12-j1. This value was very similar to the results reported in [72] at 2.45 GHz. The loss factor results showed certain variability within this aggregate, maybe due to the apparent inter-crystalline porosity observed during the petrographic analysis. The permittivity determined for the pyrite content (8-j0.3) was slightly lower to the values described in [73], presumably due to the small size of the grains dispersed through the sample.

#### 3.2.2. Anorthosite

**Figure 8:**
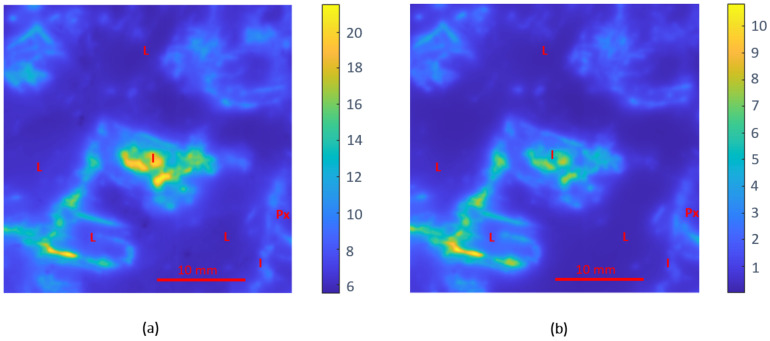
Dielectric images of the anorthosite specimen: (**a**) dielectric constant response and (**b**) loss factor results (L—labradorite plagioclase; Px—pyroxene; I—ilmenite).

Figure 8 shows the dielectric mark of the anorthosite rock specimen. The dielectric pattern of both the dielectric constant and the loss factor responses was similar to that which happened in the rock analysis described in Section 3.2.1. Figure 8a presents an area with moderate values of dielectric constant in the range of 6, which correspond to the zone of moderate–low losses in the range of 0.1, shown in Figure 7b. The rest of the rock was composed of minerals with high permittivity, both in dielectric constant and loss factor.

The area with moderate permittivity values corresponds mainly to the Na–Ca plagioclase of labradorite composition. The dielectric constant determined for this mineral is around 6-j0.1, which is in complete agreement with that reported in [71]. The dielectric response of the small quantities of pyroxene exhibited a permittivity slightly higher than that of the labradorite plagioclase, as reported in [36].

The area with high permittivity values corresponds to the ilmenite metal oxide. Previous studies in the literature reported permittivity values of this mineral with significant variability. For instance, at 2.45 GHz permittivity of ilmenite was reported to be 3.75-j0.24 in [72] and 23.6-j11.2 in [71]. Apart from the significant differences found, most of the works agree that this metal oxide have high permittivity in both the real and imaginary parts. With these permittivity values, the processing of this area with microwave energy would be excellent: the high dielectric constant values would focus the electromagnetic field in these zones of the rock, and the dielectric losses would allow microwave heating.

Indeed, there is some variability in the permittivity values of the minerals analyzed, both in our work and in those reported by other authors, probably due to the diverse composition of the minerals and their purity, the texture of the measured material (micro- or macro-crystals, powder) [71,77], the influence of the surrounding components, or the uncertainties of the different methods [36]. Nevertheless, our system has been shown to agree well with the results reported in most of the previously published studies, thus validating the applicability of the non-contact near-field microwave microscope developed for the dielectric characterization of rocks, as well as for identifying and spotting the minerals that compose them.

## 4. Conclusions

In this work, we describe the use of a near-field scanning microwave microscope as a new device for the non-contact dielectric characterization of rock specimens at the micrometer scale.

The measurement instrument includes an in-house microwave reflectometer to allow autonomous performance and additional hardware and software elements to make the measurement procedure simple and straightforward.

To allow contactless loss factor measurements, we developed a near-field sensor with enhanced quality factor sensitivity and, thus, increased loss factor resolution. The response of this near-field microwave cavity as a function of the tip-to-sample distance was modeled through a combination of the classical MCPT with an image charge theory. A comparative measurement campaign of dielectric materials with other well-established instruments was carried out to validate the proposed measurement technique. The dielectric constant accuracy was within the range of 3%, and the error in the loss factor was constrained below 10% within the range 10^−2^ to 10^1^, a remarkable result for the high tip-to-sample distances employed in the measurements.

Two types of rock with felsic and mafic compositions were selected to be analyzed with the developed sensor. Dielectric maps showed that the gneiss sample had low absorption capabilities, except in specific points disseminated throughout the rock. On the other hand, the permittivity response of the anorthosite specimen depicted moderate and high dielectric losses, and hence, presents excellent capabilities to be processed under microwave irradiation. The results obtained were found to agree well with the results reported in the literature, thus validating the performance of the proposed non-contact near-field microwave microscope.

This method will allow the identification of minerals and their dielectric characterization in the actual state in which they are found in the rock under study. In addition, the developed NSMM with enhanced sensitivity could also be employed as a stand-alone tool to determine permittivity maps of planar materials at microwave frequencies in a broad range of sensing applications.

## Figures and Tables

**Table 1:** Permittivity references of the rock-forming materials of the rock specimens.

Material	Frequency (GHz)	Permittivity	Reference
k-Feldspars	2.00	5.18-j0.023	[68]
	9.37	5.12-j0.011	[69]
	9.37	4.75-j0.044	[69]
Biotite	1.00	5.9-j0.002	[70]
Plagioclase (Labradorite)	2.45	6.01-j0.09	[71]
Plagioclase	2.45	3.49-j0.020	[37]
Plagioclase (albite)	2.45	5.62-j0.039	[36]
Pyroxene (augite)	2.45	6.80-j0.182	[36]
Hornblende	2.45	14.45-j0.324	[36]
	2.45	8.91-j0.233	[37]
	2.45	7.37-j0.026	[71]
Magnetite	2.45	14.5-j2.5	[72]
Pyrite (Shanxi Lu′an)	2.45	8-j0.75	[73]
Ilmenite	2.45	3.75-j0.24	[72]
	2.45	23.6-j11.2	[71]

**Table 2:** Dielectric properties: results of reference materials and associated standard deviations.

Material	Dielectric Constant							Loss Factor						
	g (µm)			Mean	Std Dev	Reference	Error (%)	g (µm)			Mean	Std Dev	Reference	Error (%)
	100	150	200					100	150	200				
Air	1.00	1.00	1.00	1.00	0.00	1.00	0.00	0.000	0.000	0.000	0.000	0.000	0.000	0.00
Rexolite	2.55	2.57	2.57	2.56	0.01	2.53 ± 0.05	1.23	0.000	0.002	0.007	0.003	0.003	0.001 ± 0.000	>10
Acetal	2.88	2.90	2.92	2.90	0.02	2.96 ± 0.05	2.06	0.105	0.113	0.123	0.114	0.009	0.120 ± 0.006	5.65
PVC	3.09	3.07	3.12	3.09	0.03	3.09 ± 0.06	0.11	0.024	0.027	0.031	0.027	0.004	0.025 ± 0.001	9.75
Macor	5.69	5.63	5.66	5.66	0.03	5.68 ± 0.11	0.35	0.020	0.026	0.031	0.026	0.005	0.024 ± 0.001	7.44
Alumina	8.97	9.10	8.93	9.00	0.09	8.94 ± 0.18	0.68	0.012	0.025	0.020	0.019	0.006	0.006 ± 0.000	>10
SiC	14.53	14.74	14.69	14.66	0.11	14.53 ± 0.41	2.20	2.134	2.077	2.128	2.113	0.031	2.092 ± 0.105	1.09
Temex E41030	29.19	28.32	29.56	29.02	0.64	28.28 ± 0.56	2.63	0.068	0.000	0.130	0.066	0.065	0.001 ± 0.000	>10
Temex E5980	69.88	72.19	65.79	69.28	3.24	67.25 ± 1.34	3.02	0.000	0.000	1.014	0.338	0.585	0.018 ± 0.006	>10

## References

[1] Susilowati Y., Rahyuwibowo H., Mengko T.R. Characteristic of interference color in rock forming mineral images. Proceedings of the Asia-Pacific Conference on Circuits and Systems, APCCAS.

[2] Povarov V.G., Kopylova T.N., Sinyakova M.A., Rudko V.A. (2021). Quantitative determination of trace heavy metals and selected rock-forming elements in porous carbon materials by the X-ray fluorescence method. ACS Omega.

[3] Zhou X., Liu D., Bu H., Deng L., Liu H., Yuan P., Du P., Song H. (2018). XRD-based quantitative analysis of clay minerals using reference intensity ratios, mineral intensity factors, Rietveld, and full pattern summation methods: A critical review. Solid Earth Sci..

[4] Senesi G.S. (2014). Laser-Induced Breakdown Spectroscopy (LIBS) applied to terrestrial and extraterrestrial analogue geomaterials with emphasis to minerals and rocks. Earth Sci. Rev..

[5] El Haddad J., de Lima Filho E.S., Vanier F., Harhira A., Padioleau C., Sabsabi M., Wilkie G., Blouin A. (2019). Multiphase mineral identification and quantification by laser-induced breakdown spectroscopy. Miner. Eng..

[6] Lanari P., Vho A., Bovay T., Airaghi L., Centrella S. (2019). Quantitative compositional mapping of mineral phases by electron probe micro-analyser. Geol. Soc. Spec. Publ..

[7] Srivastava P.K., Krishna A.P., Jawed S., Sarkhel P. (2020). Quantitative minerological analysis of some granite rocks of deoghar jharkhand. Earth Sci. Res..

[8] Zhang Y., Li M., Han S., Ren Q., Shi J. (2019). Intelligent identification for rock-mineral microscopic images using ensemble machine learning algorithms. Sensors.

[9] Köse C., Alp I., Ikibaş C. (2012). Statistical methods for segmentation and quantification of minerals in ore microscopy. Miner. Eng..

[10] Aligholi S., Khajavi R., Razmara M. (2015). Automated mineral identification algorithm using optical properties of crystals. Comput. Geosci..

[11] Asmussen P., Conrad O., Günther A., Kirsch M., Riller U. (2015). Semi-automatic segmentation of petrographic thin section images using a “seeded-region growing algorithm” with an application to characterize wheathered subarkose sandstone. Comput. Geosci..

[12] Izadi H., Sadri J., Bayati M. (2017). An intelligent system for mineral identification in thin sections based on a cascade approach. Comput. Geosci..

[13] Liu C., Li M., Zhang Y., Han S., Zhu Y. (2019). An enhanced rock mineral recognition method integrating a deep learning model and clustering algorithm. Minerals.

[14] Dąbrowska S., Chudoba T., Wojnarowicz J., Łojkowski W. (2018). Current trends in the development of microwave reactors for the synthesis of nanomaterials in laboratories and industries: A review. Crystals.

[15] Priecel P., Lopez-Sanchez J.A. (2019). Advantages and limitations of microwave reactors: From chemical synthesis to the catalytic valorization of biobased chemicals. ACS Sustain. Chem. Eng..

[16] Llompart M., Celeiro M., Dagnac T. (2019). Microwave-assisted extraction of pharmaceuticals, personal care products and industrial contaminants in the environment. TrAC Trends Anal. Chem..

[17] Wei W., Shao Z., Zhang Y., Qiao R., Gao J. (2019). Fundamentals and applications of microwave energy in rock and concrete processing—A review. Appl. Therm. Eng..

[18] Kahraman S., Canpolat A.N., Fener M. (2020). The influence of microwave treatment on the compressive and tensile strength of igneous rocks. Int. J. Rock Mech. Min. Sci..

[19] Lu G.M., Feng X.T., Li Y.H., Zhang X. (2019). The microwave-induced fracturing of hard rock. Rock Mech. Rock Eng..

[20] Wang J.P., Jiang T., Liu Y.J., Xue X.X. (2019). Influence of microwave treatment on grinding and dissociation characteristics of vanadium titano-magnetite. Int. J. Miner. Metall. Mater..

[21] Guo L., Lan J., Du Y., Zhang T.C., Du D. (2020). Microwave-enhanced selective leaching of arsenic from copper smelting flue dusts. J. Hazard. Mater..

[22] López-Buendía A.M., Guillem C., Cuevas J.M., Mateos F., Montoto M. (2013). Natural stone reinforcement of discontinuities with resin for industrial processing. Eng. Geol..

[23] Krupka J. (2006). Frequency domain complex permittivity measurements at microwave frequencies. Meas. Sci. Technol..

[24] García-Baños B., Cuesta-Soto F., Griol A., Catalá-Civera J.M., Pitarch J. (2006). Enhancement of sensitivity of microwave planar sensors with EBG structures. IEEE Sens. J..

[25] Pitarch J., Contelles-Cervera M., Pẽaranda-Foix F.L., Catalá-Civera J.M. (2006). Determination of the permittivity and permeability for waveguides partially loaded with isotropic samples. Meas. Sci. Technol..

[26] Pérez-Campos R., Fayos-Fernández J., Lozano-Guerrero A.J., Martínez-González A., Monzó-Cabrera J., Mediavilla I., Peña-Carro D., Esteban-Pascual L.S. (2020). Permittivity measurements for cypress and rockrose biomass versus temperature, density, and moisture content. Sensors.

[27] Neira L.M., Mays R.O., Sawicki J.F., Schulman A., Harter J., Wilke L.G., Behdad N., Van Veen B.D., Hagness S.C. (2020). A pilot study of the impact of microwave ablation on the dielectric properties of breast tissue. Sensors.

[28] González-Teruel J.D., Jones S.B., Soto-Valles F., Torres-Sánchez R., Lebron I., Friedman S.P., Robinson D.A. (2020). Dielectric spectroscopy and application of mixing models describing dielectric dispersion in clay minerals and clayey soils. Sensors.

[29] Oliveira J.G.D., Junior J.G.D., Pinto E.N.M.G., Neto V.P.S., D’Assunção A.G. (2020). A new planar microwave sensor for building materials complex permittivity characterization. Sensors.

[30] Gutierrez-Cano J.D., Plaza-Gonzalez P., Canos A.J., Garcia-Banos B., Catala-Civera J.M., Penaranda-Foix F.L. (2020). A new stand-alone microwave instrument for measuring the complex permittivity of materials at microwave frequencies. IEEE Trans. Instrum. Meas..

[31] Lu G., Zhou J., Li Y., Zhang X., Gao W. (2020). The influence of minerals on the mechanism of microwave-induced fracturing of rocks. J. Appl. Geophys..

[32] Deyab S.M., Rafezi H., Hassani F., Kermani M., Sasmito A.P. (2021). Experimental investigation on the effects of microwave irradiation on kimberlite and granite rocks. J. Rock Mech. Geotech. Eng..

[33] Lovás M., Kováčová M., Dimitrakis G., Čuvanová S., Znamenáčková I., Jakabský Š. (2010). Modeling of microwave heating of andesite and minerals. Int. J. Heat Mass Transf..

[34] Pittella E., Angrisani L., Cataldo A., Piuzzi E., Fabbrocino F. (2020). Embedded split ring resonator network for health monitoring in concrete structures. IEEE Instrum. Meas. Mag..

[35] D’Alvia L., Pittella E., Rizzuto E., Piuzzi E., Del Prete Z. (2021). A portable low-cost reflectometric setup for moisture measurement in cultural heritage masonry unit. Meas. J. Int. Meas. Confed..

[36] Zheng Y.L., Zhao X.B., Zhao Q.H., Li J.C., Zhang Q.B. (2020). Dielectric properties of hard rock minerals and implications for microwave-assisted rock fracturing. Geomech. Geophys. Geo-Energy Geo-Resour..

[37] Zheng Y., Sun T. (2021). A method to derive the dielectric loss factor of minerals from microwave heating rate tests. Meas. J. Int. Meas. Confed..

[38] Anlage S.M., Talanov V.V., Schwartz A.R., Kalinin S., Gruverman A. (2007). Principles of near-field microwave microscopy. Scanning Probe Microscopy: Electrical and Electromechanical Phenomena at the Nanoscale.

[39] Vlahacos C.P., Black R.C., Anlage S.M., Amar A., Wellstood F.C. (1996). Near-field scanning microwave microscope with 100 μm resolution. Appl. Phys. Lett..

[40] Gregory A.P., Blackburn J.F., Lees K., Clarke R.N., Hodgetts T.E., Hanham S.M., Klein N. (2016). Measurement of the permittivity and loss of high-loss materials using a Near-Field Scanning Microwave Microscope. Ultramicroscopy.

[41] Ramzi M.R., Abou-Khousa M., Prayudi I. (2017). Near-field microwave imaging using open-ended circular waveguide probes. IEEE Sens. J..

[42] Xie Z., Li Y., Sun L., Wu W., Cao R., Tao X. (2020). A simple high-resolution near-field probe for microwave non-destructive test and imaging. Sensors.

[43] Imtiaz A., Wallis T.M., Kabos P. (2014). Near-field scanning microwave microscopy: An emerging research tool for nanoscale metrology. IEEE Microw. Mag..

[44] Paulson C.A., Van Der Weide D.W., Kalinin S., Gruverman A. (2007). Near-field high-frequency probing. Scanning Probe Microscopy.

[45] Tabib-Azar M., Zhang T., LeClair S.R. (2002). Self-oscillating evanescent microwave probes for nondestructive evaluations of materials. IEEE Trans. Instrum. Meas..

[46] Wu B.Y., Sheng X.Q., Fabregas R., Hao Y. (2017). Full-wave modeling of broadband near field scanning microwave microscopy. Sci. Rep..

[47] Gao C., Xiang X.D. (1998). Quantitative microwave near-field microscopy of dielectric properties. Rev. Sci. Instrum..

[48] Gao C., Hu B., Takeuchi I., Chang K.S., Xiang X.D., Wang G. (2005). Quantitative scanning evanescent microwave microscopy and its applications in characterization of functional materials libraries. Meas. Sci. Technol..

[49] Cheng H.F., Chen Y.C., Lin I.N. (2006). Evanescent microwave probe study on dielectric properties of materials. J. Eur. Ceram. Soc..

[50] Kimber D.P., Pullar R.C., Alford N.M.N. (2008). The effects of dielectric loss and tip resistance on resonator Q of the scanning evanescent microwave microscopy (SEMM) probe. Meas. Sci. Technol..

[51] Geaney S., Cox D., Hönigl-Decrinis T., Shaikhaidarov R., Kubatkin S.E., Lindström T., Danilov A.V., de Graaf S.E. (2019). Near-field scanning microwave microscopy in the single photon regime. Sci. Rep..

[52] Tselev A., Yu P., Cao Y., Dedon L.R., Martin L.W., Kalinin S.V., Maksymovych P. (2016). Microwave a.c. conductivity of domain walls in ferroelectric thin films. Nat. Commun..

[53] Gangwar A.K., Kanika, Kedawat G., Papanai G.S., Gupta B.K. (2019). Single excitable dual emissive novel luminescent pigment to generate advanced security features for anti-counterfeiting applications. J. Mater. Chem. C.

[54] Tai T., Ghamsari B.G., Bieler T.R., Tan T., Xi X.X., Anlage S.M. (2014). Near-field microwave magnetic nanoscopy of superconducting radio frequency cavity materials. Appl. Phys. Lett..

[55] Hoffmann J., Gramse G., Niegemann J., Zeier M., Kienberger F. (2014). Measuring low loss dielectric substrates with scanning probe microscopes. Appl. Phys. Lett..

[56] Farina M., Jin X., Fabi G., Pavoni E., Di Donato A., Mencarelli D., Morini A., Piacenza F., Al Hadi R., Zhao Y. (2019). Inverted scanning microwave microscope for in vitro imaging and characterization of biological cells. Appl. Phys. Lett..

[57] Monti T., Tselev A., Udoudo O., Ivanov I.N., Dodds C., Kingman S.W. (2016). High-resolution dielectric characterization of minerals: A step towards understanding the basic interactions between microwaves and rocks. Int. J. Miner. Process..

[58] Gutiérrez-Cano J.D., Catalá-Civera J.M., Plaza-González P.J., Peñaranda-Foix F.L. (2021). Detection of anti-counterfeiting markers through permittivity maps using a micrometer scale near field scanning microwave microscope. Sensors.

[59] Caja M.Á., Peña A.C., Campos J.R., Diego L.G., Tritlla J., Bover-Arnal T., Martín-Martín J.D. Image processing and machine learning applied to lithology identification, classification and quantification of thin section cutting samples. Proceedings of the SPE Annual Technical Conference and Exhibition.

[60] Acosta I.C.C., Khodadadzadeh M., Tusa L., Ghamisi P., Gloaguen R. (2019). A Machine learning framework for drill-core mineral mapping using hyperspectral and high-resolution mineralogical data fusion. IEEE J. Sel. Top. Appl. Earth Obs. Remote Sens..

[61] Harraden C.L., Cracknell M.J., Lett J., Berry R.F., Carey R., Harris A.C. (2019). Automated core logging technology for geotechnical assessment: A study on core from the Cadia East porphyry deposit. Econ. Geol..

[62] Metaxas A.C. (1991). Microwave heating. Power Eng. J..

[63] Kajfez D. (1994). Linear fractional curve fitting for measurement of high Q factors. IEEE Trans. Microw. Theory Tech..

[64] Kajfez D. (1994). Q-Factor.

[65] Kleismit R.A., Kazimierczuk M.K., Kozlowski G. (2006). Sensitivity and resolution of evanescent microwave microscope. IEEE Trans. Microw. Theory Tech..

[66] Altschuler H.M., Sucher M., Fox J. (1963). Dielectric constant. Handbook of Microwave Measurements.

[67] Khanna S.K., Ehrenfreund E., Garito A.F., Heeger A.J. (1974). Microwave properties of high-purity tetrathiofulvalene-tetracyanoquinodimethan (TTF-TCNQ). Phys. Rev. B.

[68] Mao W., Wu L., Qi Y. (2020). Impact of compressive stress on microwave dielectric properties of feldspar dpecimen. IEEE Trans. Geosci. Remote Sens..

[69] Zheng Y., Wang S., Feng J., Ouyang Z., Li X. (2005). Measurement of the complex permittivity of dry rocks and minerals: Application of polythene dilution method and Lichtenecker’s mixture formulae. Geophys. J. Int..

[70] Church R.H., Webb W.E., Salsman J.B. (1988). Dielectric Properties of Low-Loss Minerals.

[71] Nelson S., Lindroth D., Blake R. (1989). Dielectric properties of selected and purified minerals at 1 to 22 GHz. J. Microw. Power Electromagn. Energy.

[72] Harrison P.C. (1997). A Fundamental Study of the Heating Effect of 2.45 GHz Microwave Radiation on Minerals.

[73] Zhang B., Yan G., Zhao Y., Zhou C., Lu Y. (2017). Coal pyrite microwave magnetic strengthening and electromagnetic response in magnetic separation desulfurization process. Int. J. Miner. Process..

[74] Penaranda-Foix F.L., Janezic M.D., Catala-Civera J.M., Canos A.J. (2012). Full-wave analysis of dielectric-loaded cylindrical waveguides and cavities using a new four-port ring network. IEEE Trans. Microw. Theory Tech..

[75] Duarte F.A., Waechter S.R., Pedrotti M.F., Pardinho R.B., Flores E.M.M., Barin J.S. (2020). Microwave-induced combustion in disposable vessels: A novel perspective for sample digestion. Anal. Chem..

[76] Lu G.-M., Li Y.-H., Hassani F., Zhang X. (2017). The influence of microwave irradiation on thermal properties of main rock-forming minerals. Appl. Therm. Eng..

[77] Pandey C.S., Jodlauk S., Schreuer J. (2011). Correlation between dielectric properties and chemical composition of the tourmaline single crystals. Appl. Phys. Lett..

